# Supplementary material for: Splice-Junction-Based Mapping of Alternative Isoforms in the Human Proteome
Source: Cell Rep. Author manuscript; Available in PMC 2020 Jan 15. (PMC6961840; doi:10.1016/j.celrep.2019.11.026)
Supplement: 3 [file NIHMS1546469-supplement-3.zip › DF2/PXD0010154/Liver-160-Q96CW1-SQTKEEQSQITSQVTGQIGWR.pdf]

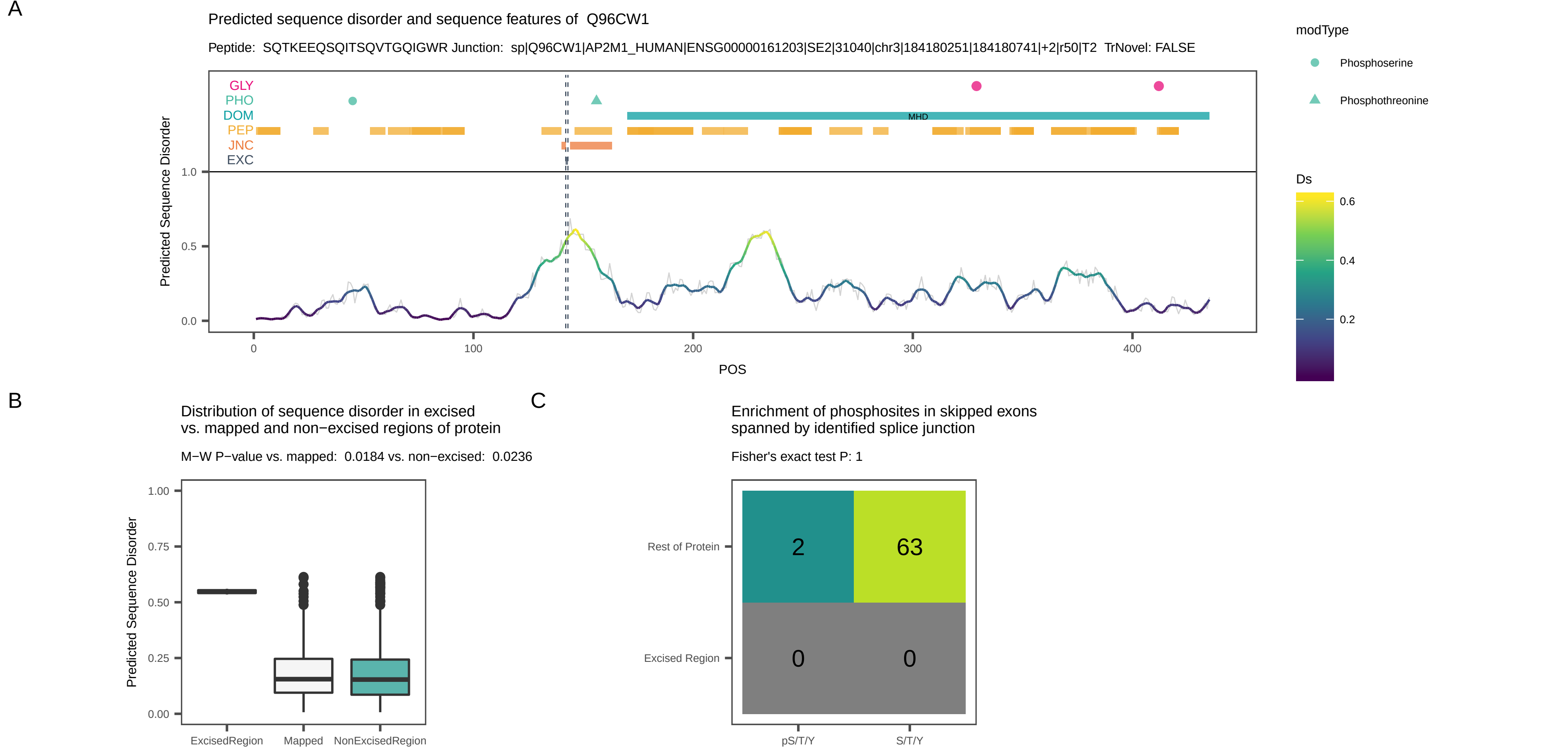
A
Predicted sequence disorder and sequence features of Q96CW1
Peptide: SQTKEEQSQITSQVTGQIGWR Junction: sp|Q96CW1|AP2M1_HUMAN|ENSG00000161203|SE2|31040|chr3|184180251|184180741|+2|r50|T2 TrNovel: FALSE
GLY
PHO
DOM
PEP
JNC
EXC
MHD
Predicted Sequence Disorder
1.0
0.5
0.0
0
100
200
300
400
POS
modType
Phosphoserine
Phosphothreonine
Ds
0.6
0.4
0.2
B
Distribution of sequence disorder in excised vs. mapped and non−excised regions of protein
M−W P−value vs. mapped: 0.0184 vs. non−excised: 0.0236
Predicted Sequence Disorder
1.00
0.75
0.50
0.25
0.00
ExcisedRegion
Mapped
NonExcisedRegion
C
Enrichment of phosphosites in skipped exons spanned by identified splice junction
Fisher's exact test P: 1
Rest of Protein
Excised Region
2
63
0
0
pS/T/Y
S/T/Y
